# A tutorial on the what, why, and how of Bayesian analysis: Estimating mood and anxiety disorder prevalence using a Canadian data linkage study

**DOI:** 10.1371/journal.pmen.0000253

**Published:** 2025-02-26

**Authors:** Myanca Rodrigues, Jordan Edwards, Tea Rosic, Yanchen Wang, Jhalok Ronjan Talukdar, Saifur R. Chowdhury, Sameer Parpia, Glenda Babe, Claire de Oliveira, Richard Perez, Zainab Samaan, Lehana Thabane

**Affiliations:** 1 Department of Health Research Methods, Evidence, and Impact, McMaster University, Hamilton, Canada; 2 Biostatistics Unit, St. Joseph’s Healthcare Hamilton, Hamilton, Canada; 3 Offord Centre for Child Studies, McMaster University, Hamilton, Canada; 4 Department of Psychiatry, University of Ottawa, Ottawa, Canada; 5 Children’s Hospital of Eastern Ontario Research Institute, Ottawa, Canada; 6 Department of Oncology, McMaster University, Hamilton, Canada; 7 The Centre for Addiction and Mental Health, Toronto, Canada; 8 ICES, Hamilton, Canada; 9 Institute of Health Policy, Management and Evaluation, University of Toronto, Toronto, Canada; 10 Department of Psychiatry and Behavioural Neurosciences, McMaster University, Hamilton, Canada; 11 Mood Disorders Program, St. Joseph’s Healthcare Hamilton, Hamilton, Canada; 12 Department of Psychiatry, Queen’s University, Kingston, Canada; 13 Faculty of Health Sciences, University of Johannesburg, Johannesburg, South Africa; Association for Socially Applicable Research (ASAR), INDIA

## Abstract

Bayesian analyses offer a robust framework for integrating data from multiple sources to better inform population-level estimates of disease prevalence. This methodological approach is particularly suited to instances where data from observational studies is linked to administrative health records, with the capacity to advance our understanding of psychiatric disorders. The objective of our paper was to provide an introductory overview and tutorial on Bayesian analysis for primary observational studies in mental health research. We provided: (i) an overview of Bayesian statistics, (ii) the utility of Bayesian methods for psychiatric epidemiology, (iii) a tutorial example of a Bayesian approach to estimating the prevalence of mood and/or anxiety disorders in observational research, and (iv) suggestions for reporting Bayesian analyses in health research.

## Author summary

Mental health conditions, like anxiety and depression, are common and can greatly impact people’s lives. Knowing how many people are affected is important for planning healthcare services and improving treatment. However, combining information from different sources, such as health surveys and medical records, may be challenging with traditional methods.

In this study, we demonstrate how Bayesian analysis, a statistical approach, may address these challenges. Using a real-world example from a study on people with opioid use disorder, we demonstrate how this method provides more accurate estimates of how common mental health conditions are. Our results highlight that Bayesian analysis effectively integrates data from different sources to produce reliable prevalence estimates.

This tutorial is the first step-by-step guide to help researchers apply Bayesian analysis in mental health research. By making this method more accessible, we aim to improve how mental illness is measured. This can ultimately help healthcare providers make better decisions about resource allocation and care for people with mental health needs.

**What is new?**

**What is the issue and why is it important?**

- Estimating the prevalence of psychiatric disorders in observational studies often relies on frequentist methods, which pose limitations in integrating multiple data sources and providing direct probability statements.
- Bayesian methods address these limitations, offering a more accurate approach for combining multiple data sources and improving prevalence estimates in psychiatric epidemiology.

**What does this study add?**

- This is the first step-by-step tutorial offering guidance on how to conduct a Bayesian analysis in primary mental health research to obtain more accurate prevalence estimates of mood and anxiety disorders.

**What are the implications?**

- Bayesian methods provide flexible, data-informed estimates that can enhance decision-making in psychiatric research.
- Mental health researchers should incorporate Bayesian methods when using multiple data sources to refine prevalence estimates and improve the reliability of their findings.

## Background

In epidemiological research, estimating population-level parameters, such as disease prevalence, is essential, particularly for informing health service planning in healthcare systems. Multiple data sources, including administrative health records, self-reported diagnostic tools, and structured surveys, are commonly used for this purpose. However, each data source has its own limitations: administrative health records often lack detailed clinical information, self-reported tools may be prone to recall bias, and structured surveys are limited by sample size and representativeness. These limitations contribute to discrepancies in prevalence estimates across sources, making it challenging to determine an accurate and reliable measure of disease prevalence. [1–3]

Statistical methods that integrate estimates from multiple sources can overcome these challenges by leveraging the strengths of each type of data. Combining data sources provides a more robust and comprehensive understanding of the true prevalence of a condition, which is critical for effective healthcare planning and resource allocation. [4–6]

Bayesian analysis offers a framework for integrating information across multiple data sources to arrive at an accurate estimate of a parameter, e.g., the true prevalence of a disease. [7] This method has the capacity to advance epidemiology research, particularly in the field of psychiatry, where observational data has been linked to administrative health data for estimating disease prevalence. [5]

While Bayesian analyses are commonly used in meta-research, i.e., meta-analysis and network meta-analysis,[8] these analytic techniques are not the aim of our tutorial. Our tutorial focuses on Bayesian methods for primary observational studies, and we encourage readers interested in Bayesian statistics for meta-research to refer to the references cited for further reading. [8–12]

The objective of our paper is to provide an introductory overview of how to approach Bayesian analyses in psychiatric epidemiology research, with a particular focus on estimating disease prevalence from observational studies. We followed guidance from other tutorials in health research, and provided an overview of the “what”, “why”, and “how” for conducting Bayesian analyses in mental health research. [13–16] We first summarized Bayesian methodology, and defined commonly-used terms (*what*). [17–19] We subsequently detailed the utility of Bayesian methods for mental health research (*why*). [7,20] We then provided an example of a Bayesian analysis from our research, and concluded with interpreting and reporting Bayesian analyses (*how*). [21–24]

## Overview of Bayesian statistics

### What are Bayesian statistics?

**Fig 1:**
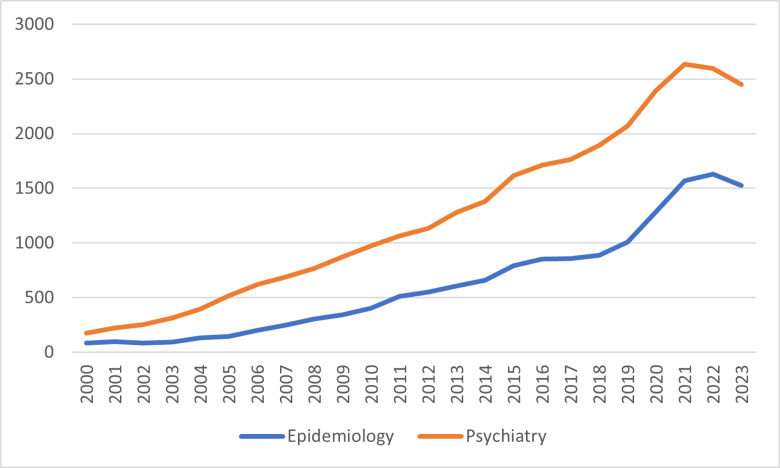
Number of PubMed indexed publications reporting Bayesian analyses. PubMed search query 1: ((epidemiology) AND ((bayesian analysis) OR (bayesian statistics))). PubMed search query 2: ((((psychiatry) OR (mental health)) OR (substance use)) AND (bayesian analysis)) OR (bayesian statistics).

Bayesian statistics is a powerful framework for inference and decision-making that has gained increasing popularity in epidemiological research (*see* Fig 1). [25] The term “Bayesian” originates from the work of Thomas Bayes, an 18^th^ century mathematician, whose theorem forms the foundation of Bayesian inference. Bayes’ theorem helps us understand how new evidence affects our beliefs about a hypothesis. [26,27]

### Bayes’ theorem

Bayes’ theorem describes how to update our beliefs about a hypothesis or parameter (*θ* ) when new data (*D*) is observed. It is expressed as: $P(\theta |D)=P(D|\theta )*P\left(\theta \right)/P\left(D\right)$, where:

- P(θ|D) is the posterior distribution, representing the updated belief about the parameter *θ* after observing the data *D*.
- P(D|θ) is the likelihood function, describing the probability of observing the data *D* given the parameter *θ*.
- $P\left(\theta \right)$ is the prior distribution, representing the initial belief about the parameter *θ* before observing the data.
- $P\left(D\right)$ is the marginal likelihood or evidence, which acts as a normalizing constant.

Bayes’ theorem tells us that the posterior probability of a hypothesis being true, given the observed data depends on several factors: (i) the likelihood of the data under the assumption that the hypothesis is true, (ii) the degree of our prior belief in the hypothesis before examining the data, and (iii) the likelihood of observing the data irrespective of the hypothesis. [26,27] In essence, Bayesian statistics enables researchers to incorporate prior beliefs or knowledge about a hypothesis with the observed data from their ongoing research, allowing for a more comprehensive understanding and analysis of the given hypothesis. [28–30]

## Differences between Frequentist and Bayesian methods: inference and selected advantages and disadvantages

The primary distinction between Frequentist (classical) statistics and Bayesian statistics lies in their approach to inference. Frequentist methods focus on *p*-values and null hypothesis significance testing, interpreting probability as the frequency of events in repeated trials. In contrast, Bayesian methods interpret probability as a degree of belief, quantifying uncertainty through probability distributions. [31] This allows Bayesian analysis to provide direct probability statements about parameters, enabling intuitive interpretation of research findings, particularly in clinical trials and observational studies.

### Applications to experimental and observational research

#### Clinical trials.

In clinical trials, Bayesian methods offer clear advantages in situations with limited sample sizes or incomplete data. For example, consider a trial comparing a new drug to a placebo for reducing blood pressure. A Frequentist analysis might conclude “no significant difference” if the p-value exceeds 0.05, without quantifying the likelihood of the drug’s effectiveness. In contrast, a Bayesian approach integrates trial data with prior information (e.g., expert knowledge or previous studies) to calculate the posterior probability that the drug is superior. [24,32,33]

This probabilistic interpretation enhances decision-making by directly addressing clinical benefit. Additionally, Bayesian models allow adaptive trial designs, where data can be analyzed sequentially to refine estimates, potentially reducing participant burden and resource use. [27,31,34–37].

#### Observational research.

Bayesian methods are also well-suited to observational studies, where randomized control over exposures is not possible. By incorporating prior distributions that reflect established knowledge, Bayesian approaches strengthen inferences and reduce bias. For instance, in studies of mental health outcomes, integrating prior data on prevalence rates can improve precision in estimating treatment effects. However, challenges such as the subjectivity of priors and computational demands remain. Properly chosen priors and careful modeling of confounding factors are critical to ensuring robust results. [38,39]

### Advantages and disadvantages

Bayesian methods offer several advantages:

- Flexibility in handling small sample sizes and complex study designs.
- Ability to incorporate prior knowledge for more informed estimates.
- Direct probabilistic interpretation of parameters, enhancing decision-making. [27,31,34–37]

However, Bayesian analysis also has drawbacks:

- Subjectivity in selecting priors (*see definition in* Table 1), which may bias results.
- Computational intensity and the need for specialized expertise.
- Challenges in disseminating results to stakeholders unfamiliar with Bayesian interpretation. [27,38–40]

**Table 1:** Definitions of commonly-used statistical terms.

Term	Definition
Credible interval	There is a 95% probability that the true estimate would lie within the interval.
Confidence interval^1^	If this process was repeated many times, 95% of the intervals would contain the true effect.
Markov chains	A stochastic model describing a sequence of possible events in which the probability of each event depends only on the state attained in the previous event.
Monte Carlo simulation	A class of algorithms for sampling from a (complex) probability distribution.
Parameter^2^	A quantity of interest in a statistical model, often representing some aspect of the underlying population.
Posterior	The conditional probability of a parameter given the observed data, obtained through Bayes’ theorem.
Prior	A probability distribution representing the researcher’s beliefs about the parameter before observing data. May be non-informative or informative.

***Notes:***

^1^Frequentist statistics.

^2^Applicable to statistics overall, both Bayesian and frequentist

While Bayesian methods have previously relied on specialized software like WinBUGS or JAGS, there are presently more user-friendly options. For example, as we will explore in our tutorial, R offers several packages for Bayesian analyses, facilitating the use of Bayesian methodology in health research. Thus, despite the aforementioned drawbacks, Bayesian statistics remains a valuable tool for epidemiology research. We have summarized commonly used terms in Bayesian methodology in Table 1, which we will refer to in our tutorial example.

## Narrowing the focus: Why use Bayesian analysis in psychiatry research?

While Bayesian analyses serve the primary purpose of offering direct probability statements, there are three specific advantages to using Bayesian methodology in psychiatry research.

### Small sample sizes

First, empirical studies have demonstrated that mental health research is often plagued by small sample sizes, given the challenges in recruiting large cohorts when studying people with mental health disorders. For instance, research in paediatric and adolescent mental health often experiences challenges with recruiting adequately powered samples for neuroimaging studies. [41,42] Unlike frequentist approaches, which depend on the asymptotic properties of adequately-powered samples, Bayesian estimation is known to perform well with even small sizes, rendering it advantageous for use in mental health research. [40,43]

### Sequential data collection

Second, Bayesian analysis permits sequential data collection, in contrast to frequentist null-hypothesis significance testing. Bayesian methodology allows researchers to adaptively incorporate new data when available. Thus, researchers are able to continually sample new cases and integrate them into the analysis, updating the results until reaching a desired level of precision in the parameter estimates. This offers additional advantages when there is insufficient information available to plan sample sizes, with researchers being able to conduct the study until reaching a desired level of certainty in their conclusions. [44] An empirical study in child psychiatry demonstrated that the use of Bayesian analysis would have permitted definitive conclusions earlier during the conduct of many studies, i.e., with smaller sample sizes. [45] Thus, the use of Bayesian methods would have optimized resource allocation and minimized participant burden, [46,47] which are integral to managing research funds and considering the time and effort invested by vulnerable populations with mental health disorders.

### Ability to incorporate priors

Third, Bayesian analysis relies on the use of priors – knowledge regarding parameters from previous research. [48] Despite the potential subjectivity in priors and their reliance on assumptions, this is not unique to Bayesian analysis, given the assumptions underlying frequentist statistics. Moreover, with the various data sources typically used in mental health research, i.e., structured surveys and administrative health data, researchers often possess prior knowledge regarding parameters. [1–3,48] By incorporating informative priors through Bayesian methodology, particularly in a study with small sample size, researchers are able to guard against spurious findings and ensure that parameter estimates are not inflated by excluding relevant information, ultimately reducing uncertainty in the analysis. [31,34,35]

Bayesian methods, therefore, offer a coherent and intuitive way to integrate existing knowledge with new evidence, making it particularly suitable for analyzing epidemiological research which utilizes multiple data sources. [49,50]

### Predictive probability

Last, as an extension of the above use of priors, Bayesian analysis may be used in prediction and disease forecasting. Bayesian methods are able to integrate current estimates of disease prevalence with informative priors to make statistically valid predictions about disease occurrence and future spread. [51] For instance, Bayesian analyses are currently employed in the United Kingdom, where prevalence estimates from nationally-administered surveys and information regarding regional variation in social and environmental factors have been integrated to forecast mental health service demands, i.e., the anticipated need for psychosis care in England up to 2025. [52] Given that mental health disorders such as depression and anxiety are among the leading causes of global morbidity,[53] the use of Bayesian methods for disease forecasting may be especially important to advancing our understanding of disease burden, and useful in health service planning for psychiatry. [4]

### Examples of Bayesian analysis in psychiatry research

Bayesian methods have seen a marked increase in psychiatry research over the past two decades (*see* Fig 1), offering solutions to challenges like small sample sizes and complex data structures. For instance, a cohort study assessing post-traumatic stress disorder (PTSD) in burn survivors requiring mechanical ventilation faced limited recruitment and reduced statistical power. By incorporating informative priors from simulation studies, Bayesian analysis provided conclusive evidence of a negative effect of mechanical ventilation on PTSD—results that frequentist methods could not establish due to insufficient power. [40] Similarly, another cohort study on the comorbidity of mood and anxiety disorders in individuals with PTSD used informative priors based on prior findings to estimate current and lifetime comorbidity rates with greater precision compared to frequentist methods. [54] These examples highlight the ability of Bayesian methods to address data limitations, improve precision, and provide actionable insights in psychiatry research, especially when small sample sizes or incomplete data hinder traditional statistical approaches.

## Tutorial: Using Bayesian methods to estimate the prevalence of psychiatric disorders

The core of our paper is focussed on a tutorial example where we estimated the prevalence of mood and/or anxiety disorders among people with opioid use disorder in Ontario, Canada. Our previous study used frequentist methods to obtain prevalence estimates for various psychiatric comorbidities (including mood and anxiety disorders - separately) among this population using self-reported responses to a diagnostic tool only. [55] However, we aim to use Bayesian methods to integrate information from administrative health databases with the data from this observational study to obtain a more accurate prevalence estimate.

A Bayesian approach to estimating the prevalence of mood and/or anxiety disorders among people with opioid use disorder using multiple data sources: a prospective cohort study

### Background

Opioid use disorder (OUD) is a public health challenge in Canada, and claims the lives of more than twenty-one Canadians daily. [56,57] There is a higher incidence of comorbid psychiatric disorders, in particular, mood (e.g., major depressive disorder (MDD) and bipolar disorder) and anxiety (e.g., generalized anxiety disorder and panic disorder) disorders among people with OUD as compared to people without OUD. [58–61] A recent systematic review and meta-analysis has estimated that the prevalence of MDD is 36.1% (95% confidence interval (CI): 32.4, 39.7%), bipolar disorder is 8.7% (95% CI: 6.7, 10.7%), and any anxiety disorder is 29.1% (95% CI: 24.0, 33.3%) among people with OUD. [59] Comorbid mood and anxiety disorders is often associated with poorer treatment outcomes among people with OUD. [62–64] Furthermore, people with OUD who additionally have mood and anxiety disorders often experience more severe physical health [65] and social issues (i.e., incarceration and low socioeconomic status) [66] than those with OUD alone.

Although prior studies have documented the extent of comorbid mood and/or anxiety disorders among people with OUD, these estimates are often informed by single-data sources, most often data from self-reported diagnostic tools, e.g., the Beck Depression Inventory, or administrative health records. In regions with universal healthcare, administrative data captures contact with the health care system, for example, outpatient and inpatient visits for mood and anxiety disorders among people with OUD. [67] Conversely, diagnoses derived from diagnostic tools, self-reported by people with OUD enrolled in observational studies, provide insights about these psychiatric disorders for those who may experience these conditions, but not seek regional health services for management or rely on private services not captured in health service records. [68] Prior research has demonstrated that the use of either data source may respectively result in an under- or over-estimation of the true prevalence of mood and/or anxiety disorders. [5] In particular, administrative health data underestimates prevalence, while self-reported diagnostic tools and surveys may overestimate the prevalence of mood and/or anxiety disorders. [1,69]

Accurate population-based estimates of the prevalence of mood and/or anxiety disorders among people with OUD are essential for effective planning and resource allocation in health systems. [4] Clinicians caring for people with OUD require a comprehensive understanding of comorbid mental health conditions to deliver comprehensive and tailored care, address potential treatment barriers, and improve treatment outcomes. Combining data from self-reported diagnostic tools and administrative records may optimally capture the full extent of mood and/or anxiety disorders in this clinical population. [6] Bayesian analysis provides a framework for integrating these data sources, leveraging prior information from health administrative records and current self-reported data from observational research to yield accurate prevalence estimates of mood and/or anxiety disorders. [7]

Bayesian methodology has been used by a recent study, which estimated the prevalence of mood and/or anxiety disorders using structured survey data linked to Ontario health administrative databases,[6] and is suitable for addressing potential under- or over-estimations in disease prevalence from sole use of one data source. Our previous study, which estimated the prevalence of mental health disorders among people with OUD using a self-reported diagnostic tool[55], has recently been linked to Ontario health administrative databases, facilitating a Bayesian analysis.

### Objective

The primary objective of our study was to use a Bayesian analysis to obtain a more informative estimate of the combined prevalence of mood and/or anxiety disorders among people receiving treatment for OUD in Ontario, Canada. By using primary data from our cohort study, in addition to secondary data in administrative health records, we aim to derive a more informed prevalence estimate.

## Methods

### Study design and setting

We used observational data from the Genetics of Opioid Addiction (GENOA) program, a prospective single-cohort study, for our current paper. The GENOA study recruited people with OUD undergoing opioid agonist treatment (OAT) at 20 community-based outpatient clinics across Ontario, Canada between 01/06/2011 and 30/04/2017. These clinics are part of a centrally managed network run by the Canadian Addiction Treatment Centres. Ethical approval was granted by the Hamilton Integrated Research Ethics Board (HiREB) (GENOA project ID 11-056), with informed consent obtained in writing from all study participants. Further details regarding GENOA study methods have been described previously. [70–73]

We followed reporting guidance in accordance with the *Strengthening the Reporting of Observational studies in Epidemiology* (STROBE) [74] and Reporting Of Bayes Used in clinical Studies (ROBUST) guidelines. [21] Given the tutorial nature of our manuscript on Bayesian analyses, we prioritized this aspect of reporting and condensed or omitted several sections of the former checklist (*see* S1 File *STROBE checklist and* S2 File *ROBUST checklist*). Given our use of administrative health data for our priors, we also incorporated some reporting elements present in the Reporting of studies Conducted using Observational Routinely-collected Data (RECORD) guidelines as appropriate. [75] For full reporting of prospective observational studies using Bayesian methods, adherence to the STROBE guidelines is recommended, along with the necessary Bayesian reporting guidelines (*see section on reporting guidance below for more details on the latter*).

### Participants

Study participants were screened and recruited in accordance with the following inclusion criteria: males and females aged 18 years or older, with a diagnosis of OUD according to the Diagnostic and Statistical Manual of Psychiatric disorders, Fourth Edition (DSM-IV) criteria, and receiving OAT for their OUD. Participants were additionally required to provide written informed consent. Exclusion criteria included people with OUD who were unable to communicate in English. All treatment centers included in the study are centrally managed and adhere to the same management protocols.

### Data sources

Data from the GENOA study was subsequently combined with data from the Pharmacogenetics of Opioid Substitution Treatment Response (POST) project, which was an extension of the GENOA study. The inclusion criteria were similar with the following exceptions: the minimum age was reduced from 18 to 16 years of age, and recruitment was expanded to 54 clinical sites across Ontario. The POST study, which was also a prospective cohort study, enrolled participants from 01/05/2018 to 30/04/2021. Additional methods for this study have been described previously. [76–78] Both studies, conducted by the same research team, adhered to similar protocols for recruitment and data collection, ensuring the combination of cohort data without additional risks of measurement error. Ethical approval was granted by the HiREB for the POST and the data linkage projects (POST project ID 4556; ICES linkage project IDs 12602 and 12767-C). Data from GENOA and POST cohorts were merged, with duplicate enrollments excluded, retaining the most complete record for each individual (*n* = 272 excluded; *Fig A in* S3 File *Supplementary tables and figs*).

The combined cohort data were linked with the routinely collected Ontario provincial administrative health data stored at ICES (formerly known as the Institute for Clinical Evaluative Sciences). ICES, an independent non-profit research institute, operates under Ontario’s health information privacy law, allowing it to collect and analyze health care and demographic data for health system evaluation and improvement without need for consent. ICES possesses administrative health records for all individuals enrolled in Ontario’s public health insurance program (> 96%). The individual-level cohort data were linked to unique encoded identifiers at ICES using deterministic linkage with Ontario health card numbers and dates of birth. Some exclusions were made during the data linkage process, such as invalid linkage, lack of Ontario Health Insurance Program (OHIP) eligibility, or residing outside the province *(see Fig A in* S3 File *Supplementary tables and figs*). Commencing on the day of cohort enrolment, we included health records from ICES holdings five years prior to study entry, with shorter follow-up for individuals censored due to death or relocation out of the province *(n* = 43), or recruitment after April 1, 2021 (*n* = 40), as ICES data were available only up to March 31, 2022. Identifying information for participants was available to the ICES data linkage staff only during linkage and removed after successful linkage of participant-level data to administrative health records. ICES data was accessed from 01/12/2023 to 20/05/2024 to complete analyses. All database analyses were conducted in compliance with Ontario privacy legislation, authorized under section 45 of Ontario’s Personal Health Information Protection Act, without requiring review by a Research Ethics Board.. All database analyses were conducted at ICES in compliance with Ontario privacy legislation. The use of this data was authorized under section 45 of Ontario’s Personal Health Information Protection Act, which does not require review by a Research Ethics Board.

### Baseline characteristics

We collected demographic self-reported data from the GENOA study, measured at study entry, including age (in years), sex (male/female/intersex/other), marital status (single/in a relationship), and employment status (unemployed/ employed).

### Primary outcome

Our primary outcome was the combined prevalence of mood and/or anxiety disorders among GENOA study participants, as assessed using both a self-reported diagnostic tool and administrative health data. We created a binary variable to identify our primary outcome: the presence or absence of mood and/or anxiety disorders separately in each of our data sources (*see further details below*). These disorders were combined into a single prevalence estimate, i.e., people with OUD who experienced either or both of these conditions were considered to experience the outcome. Given our need to inform clinicians who care for people with OUD, it was necessary to quantify the burden of disease within this given population. Thus, we opted to examine prevalence (existing cases of disease – a measure of disease status) rather than incidence (number of new cases of a disease within a defined period). [79–81]

Clinically, mood and anxiety disorders are often assessed together due to their high rates of comorbidity and overlapping symptoms. Many people with mood disorders, such as major depressive or bipolar disorders, also experience symptoms of anxiety disorders, and vice versa. [82,83] Assessing these mental health disorders concurrently allows for a more comprehensive evaluation of mental health, which may improve clinical outcomes and treatment planning, particularly in populations with high rates of comorbidity, such as those with OUD. [84]

This methodological approach has also been followed by a study assessing the prevalence of multiple chronic conditions in Ontario,[85] and another study which used a Bayesian analysis to estimate the combined prevalence of mood and/or anxiety disorders using structured surveys and Ontario health administrative data sources. [5]

#### Diagnoses derived from self-reported diagnostic tool (observational data).

**Fig 2:**
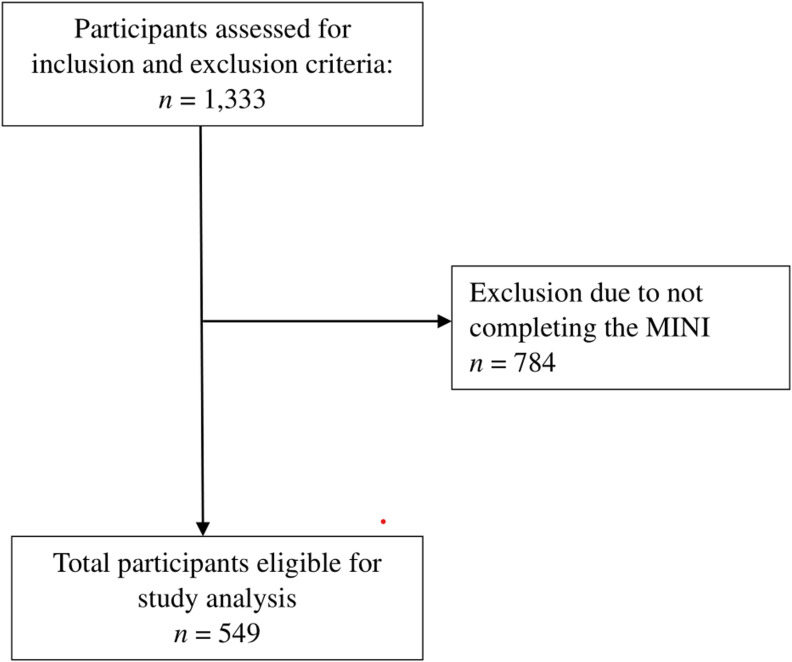
Study flow diagram for GENOA prospective cohort study. ***Abbreviations:*** GENOA: Genetics of Opioid Addiction study, MINI: Mini-International Neuropsychiatric Interview version 6.0.

The GENOA study recruited 1,333 participants. Of these, 272 were excluded due to dual enrollment in the POST study, resulting in 1,061 participants. Among these, 784 did not complete the Mini-International Neuropsychiatric Interview (MINI) version 6.0, which was discontinued due to participant burden and the time required for completion. [76] This resulted in a final sample of 549 people with OUD in the GENOA study (*see* Fig 2 *and Fig A in* S3 File *Supplementary tables and figs for details*).

The MINI 6.0 is a standardized and structured diagnostic tool widely used in research and clinical settings to ascertain psychiatric conditions,[86] including mood and anxiety disorders according to DSM-IV and the International Classification of Diseases, Tenth Revision (ICD-10) criteria. [87] The MINI has been shown to have sensitivity/specificity values of (i) 95%/84% for diagnosing MDD in a meta-analysis of diagnostic test accuracy studies, [88] and (ii) 90%/95% for the diagnosis of anxiety disorders in primary care. [89] It was administered in our study by trained study personnel. [76]

For our current study, we used the following modules of the MINI 6.0: mood disorder (MDD or bipolar affective disorder), anxiety disorder (generalized anxiety disorder, social phobia, posttraumatic stress disorder [PTSD], obsessive compulsive disorder, and panic disorder with and without agoraphobia). MINI diagnoses of mood disorders consider past and lifetime symptomatology, whereas diagnoses of anxiety disorders are based on current symptomatology in the past month. [87] Participants who met the criteria for any of the aforementioned conditions were coded as ‘1’, i.e., the presence of combined mood and/or anxiety disorders in the GENOA dataset.

#### Administrative-derived diagnoses (ICES data).

We extracted billing data on mood and anxiety disorders from linked health administrative records for the entire linked study sample, i.e., POST and GENOA cohorts, using a standardized algorithm. Similar methods have been used by other studies examining mood and anxiety disorders in Ontario. [5,85] Administrative-derived diagnoses of depression were demonstrated to have a sensitivity of 62.9% and a specificity of 93.8% in a study assessing health records in Alberta and British Columbia. [90] However, there is a paucity of evidence on these psychometric properties from Ontario-based studies on depression and/or anxiety, since OHIP codes are not sufficiently disaggregated, leading to a lack of regional validation studies on administrative-derived diagnoses of these disorders. Nonetheless, our approach has been used by a prior study examining prevalence of mood and anxiety disorders in Ontario. [5]

Cases were defined as people with OUD who met at least one of the following criteria within a 24-month period: any one inpatient hospital diagnostic code, or two or more outpatient physician billing codes (OHIP codes) using relevant ICD-9/ICD-10 codes for mood (MDD, dysthymic, or bipolar affective disorder) and anxiety (generalized anxiety disorder, phobic anxiety disorder, PTSD, other anxiety disorder, obsessive compulsive disorder, adjustment disorder and panic disorder with and without agoraphobia) disorders (*see Table A in* S3 File *Supplementary tables and figs for specific codes*). We considered a five-year lookback period prior to study entry to identify cases. Participants who met criteria for any of the aforementioned conditions were coded as ‘1’, i.e., presence of combined mood and/or anxiety disorders in the merged POST and GENOA ICES dataset.

### Data analysis

***Note:*** Definitions for bolded terms may be found in Table 1.

Descriptive statistics on baseline characteristics of GENOA study participants were summarized using frequencies and proportions for categorical data, and means and standard deviations (s.d.) for continuous data.

Our estimated **parameter** was the combined prevalence of mood and/or anxiety disorders among GENOA study participants. We first conducted a frequentist analysis to estimate the combined prevalence with a 95% **confidence interval** (CI) using the *glm* function in R, [91] with a binomial family and identity link function.

We subsequently conducted our Bayesian analyses using the *brm* function in R, [92] with an error distribution from the Bernoulli family and an identity link function. This combination is often used in modeling binary outcomes and allows for the estimation of probabilities directly. The Bernoulli distribution is suitable for modeling binary outcomes with two possible values, i.e., 0 = non-cases, 1 = cases. The identity link function ensures that the log-odds is directly related to the outcome probabilities without any transformation. [93]

We set a seed of 123 to enhance replicability for our Bayesian analyses. We estimated the marginal posterior density of our parameter using the NUTS (No-U-Turn Sampler), a **Markov Chain Monte Carlo (MCMC) technique**. The NUTS algorithm generates random samples from the posterior distributions of individual parameters by constructing a **Markov chain**, a sequence of random variables where the probability distribution of each variable depends only on the state of the preceding variable. [94] These samples are then used to compute Bayesian **credible intervals** (CrI), the Bayesian analogue to frequentist 95% confidence intervals. [28] Our **posterior** estimate represents the probability distribution of the estimated parameter, i.e., the posterior mean prevalence of mood and/or anxiety disorders among people with OUD, after integrating both prior information from the ICES datasets and observed data from the GENOA dataset.

For all Bayesian analyses, we used beta-distributed **priors**, which may be found in Table 2. These priors reflected our beliefs from prior knowledge, i.e., about the prevalence of mood and/or anxiety disorders in the ICES administrative data. [48] Our priors were in the form (α, β), where α represents the number of successes (cases – people with mood and/or anxiety disorders in the ICES databases) and β represents the number of failures (non-cases – people without either condition in the ICES databases), which is suitable for a binary outcome. [95]

**Table 2:** Selection of priors.

Type of analysis	Sample for prior (*n*)	Cases (*k*) (%)	Non-Cases (%)	α	β
Frequentist	*N/A*	*N/A*	*N/A*	*N/A*	*N/A*
Non-informative	*N/A*	*N/A*	*N/A*	1	1
Primary	GENOA participants (*n* = 1,061)	632 (59.57%)	429 (40.43%)	633	430
Sensitivity 1	POST participants (*n* = 2,369)	1394 (58.84%)	975 (41.16%)	1395	976
Sensitivity 2	GENOA + POST participants (*n* = 3,430)	2026 (59.07%)	1404 (40.93%)	2027	1405
Sensitivity 3 *(decrease iterations)*	GENOA participants (*n* = 1,061)	632 (59.57%)	429 (40.43%)	633	430
Sensitivity 4 *(increase iterations)*	GENOA participants (*n* = 1,061)	632 (59.57%)	429 (40.43%)	633	430

***Abbreviations:*** GENOA: GENetics of Opioid Addiction study, N/A: not applicable, POST: Pharmacogenetics of Opioid Substitution Treatment Response project.

Our first Bayesian analysis used a non-informative beta-distributed prior (1,1), assigning equal probability density to cases and non-cases. Non-informative priors are selected to represent a state of complete uncertainty, [96] thereby enabling the GENOA self-reported data to largely inform the posterior prevalence estimate. The remainder of our Bayesian analyses used informative priors, which were determined using the number of cases and non-cases in the ICES administrative datasets according to the formulae: α = *k* + 1, β = *n* – *k* + 1, where *k* represents the number of cases (people with mood and/or anxiety disorders), and *n* represents the total sample for that specific prior (*see* Table 2). [97] Due to the small sample of GENOA participants who completed the MINI, our primary analysis used a beta-distributed (633, 430) prior from the entire GENOA sample, i.e., both those who did and did not complete the MINI (*n* = 1,061). Given the inclusion criteria were upheld through the conduct of the study, the use of the entire sample provided us with a better-informed prior for our analysis.

For all analyses, we used summaries of the posterior mean prevalence and 95% equally tailed posterior credible intervals to interpret results.

#### Sensitivity analyses.

We conducted four sensitivity analyses to assess the robustness of our findings. Our first sensitivity analysis used a beta-distributed (1395, 976) prior from the POST sample (*n* = 2,369). Our second sensitivity analysis used a beta-distributed (2027, 1405) prior from the merged GENOA and POST sample (*n* = 3,430). As there were similarities in inclusion criteria, recruitment, and data collection between the two cohorts, we believe these priors were suitable for our sensitivity analyses, and further, provided us with larger samples.

For our analysis using a non-informative prior, primary analysis and first two sensitivity analyses, convergence was achieved with 20,000 iterations and 4 Markov chains, after a warm-up of 500 iterations and thinning of 5 iterations. Our third and fourth sensitivity analyses used the same prior as our primary analysis, but we varied the iterations and chains to gauge the effect on convergence (mixing of the Markov chains). For our third sensitivity analysis, we decreased the iterations to 50, and used 2 Markov chains, with a warm-up of 10 iterations and thinning of 1 iteration. For our fourth sensitivity analysis, we increased it to 40,000 iterations, and used 4 Markov chains, with a warm-up of 600 iterations and thinning of 10 iterations.

#### Model fit and performance.

To assess model fit and performance, we examined: (i) trace, (ii) posterior density, and (iii) autocorrelation plots for our estimated parameter for each analysis. Trace plots depict sampled parameter values over iterations, and are used to indicate convergence and well-mixed Markov chains. Density plots depict density of the estimated parameter, i.e., posterior prevalence, and convergence is reflected by unimodal and symmetric distributions. Autocorrelation measures the correlation between consecutive samples in the Markov chains, and provides another metric by which to evaluate whether our Markov chain sufficiently mimics the behaviour of an independent sample. [98,99]

We additionally inspected R-hat values, and bulk and tail effective sample sizes to assess convergence. R-hat refers to potential scale factor reduction, and compares the variance within chains to that between chains. A value of 1.0 indicates convergence, suggesting that the multiple chains used in our analysis have mixed well. The effective sample size measures chain length while taking into account chain autocorrelation. Bulk and tail effective sizes assess the quality of samples obtained from MCMC simulations, with bulk effective sample size focussing on the main body of the chain, while tail effective sample size considering the tail end of the distribution. Values of at least 1,000 suggest reliability of our analyses. [100]

#### Availability of code.

All analyses were conducted using Stan (via R),[101] and our script is available in S4 File R script (R file) and S5 File R script (Word document). Given our aim of conducting a tutorial for psychiatry researchers, we additionally conducted our primary analysis using JASP software. [102,103] JASP has a friendly graphic user interface, and is similar to Statistical Package for the Social Sciences (SPSS) software, which is commonly-used in mental health research. [104,105] We included details of our analyses in S6 File JASP guide. However, we would like to note that the Bayesian functionality in JASP is limited, and does not permit varying the number of iterations as in our third and fourth sensitivity analyses.

## Results

We screened 1,333 people with OUD for inclusion in the GENOA study. After exclusion of 272 participants, due to duplicate enrolment in the POST cohort study, and excluding 784 participants who were not administered the MINI, our final GENOA study sample included 549 people with OUD (*see* Fig 2 *and Fig A in* S3 File *Supplementary tables and figs for study flow diagrams*). Overall, 311/549 (56.6%) GENOA study participants had a mood and/or anxiety disorder, of which 174 (55.9%) were females (*see* Table 3). The mean age of study participants was 37.4 (s.d. = 10.9) years. The majority of study participants with mood and/or anxiety disorders were single (220/311, 70.7%), and unemployed (217/311, 69.8%).

**Table 3:** Baseline demographics of GENOA cohort who completed MINI.

Demographic or clinical factor	No mood and/or anxiety disorder (*n* = 238)	Mood and/or anxiety disorder (*n* = 311)
**Sex**		
Male	162 (68.1%)	137 (44.1%)
Female	76 (31.9%)	174 (55.9%)
**Age** (*years*, mean, s.d.)	40.1 (11.4)	37.4 (10.9)
**Marital status**		
Single	157 (66.0%)	220 (70.7%)
Married or common-law	81 (34.0%)	91 (29.3%)
**Employment status**		
Unemployed	131 (55.0%)	217 (69.8%)
Employed	107 (45.0%)	94 (30.2%)

***Abbreviations:*** GENOA: Genetics of Opioid Addiction study, MINI: Mini-International Neuropsychiatric Interview version 6.0, s.d.: standard deviation.

The results of our analyses may be found in Table 4. Our frequentist analysis indicates that the prevalence of mood and/or anxiety disorders is 56.6% (95% CI: 52.5, 60.8%). Thus, if this process was repeated several times, 95% of the above confidence intervals would contain the true prevalence. Our non-informative Bayesian analysis obtains a similar prevalence mean of 57.0% (CrI: 52, 61%). The difference lies in interpretation, i.e., there is a 95% probability that the true prevalence of mood and/or anxiety disorders among people with OUD lies between 52.0 and 61.0%, with the estimated intercept of 57.0%.

**Table 4:** Prevalence of mood and/or anxiety disorder in GENOA cohort.

Analysis	Prevalence (95% CI or CrI)	SE	R hat	Bulk ESS	Tail ESS
Frequentist	56.6% (52.5, 60.8%)	0.021	*N/A*	*N/A*	*N/A*
Non-informative	57.0% (52.0, 61.0%)	0.02	1.0	14,444	14,438
Primary	59.0% (56.0, 61.0%)	0.01	1.0	13,676	14,089
Sensitivity 1	58.0% (57.0, 60.0%)	0.01	1.0	14,716	14,626
Sensitivity 2	59.0% (57.0, 60.0%)	0.01	1.0	15,247	14,444
Sensitivity 3	59.0% (57.0, 60.0%)	0.01	1.05	37	48
Sensitivity 4	59.0% (56.0, 61.0%)	0.01	1.0	16,030	15,623

***Abbreviations:*** CI: confidence interval (frequentist only), CrI: credible interval (all other – Bayesian – analyses), ESS: effective sample size, N/A: not applicable, SE: standard error.

Findings from our primary analysis indicate that there is a 95% probability that the true prevalence of mood and/or anxiety disorders among people with OUD lies between 56.0 and 61.0%, with the estimated posterior mean at 59.0%. We obtained the same prevalence estimate when using JASP software (*see* S6 File *JASP guide*). Our first and second sensitivity analyses are similar to our primary analysis, in estimated intercepts, with slightly narrower credible intervals, owing to larger sample sizes of the priors used in these analyses.

**Fig 3:**
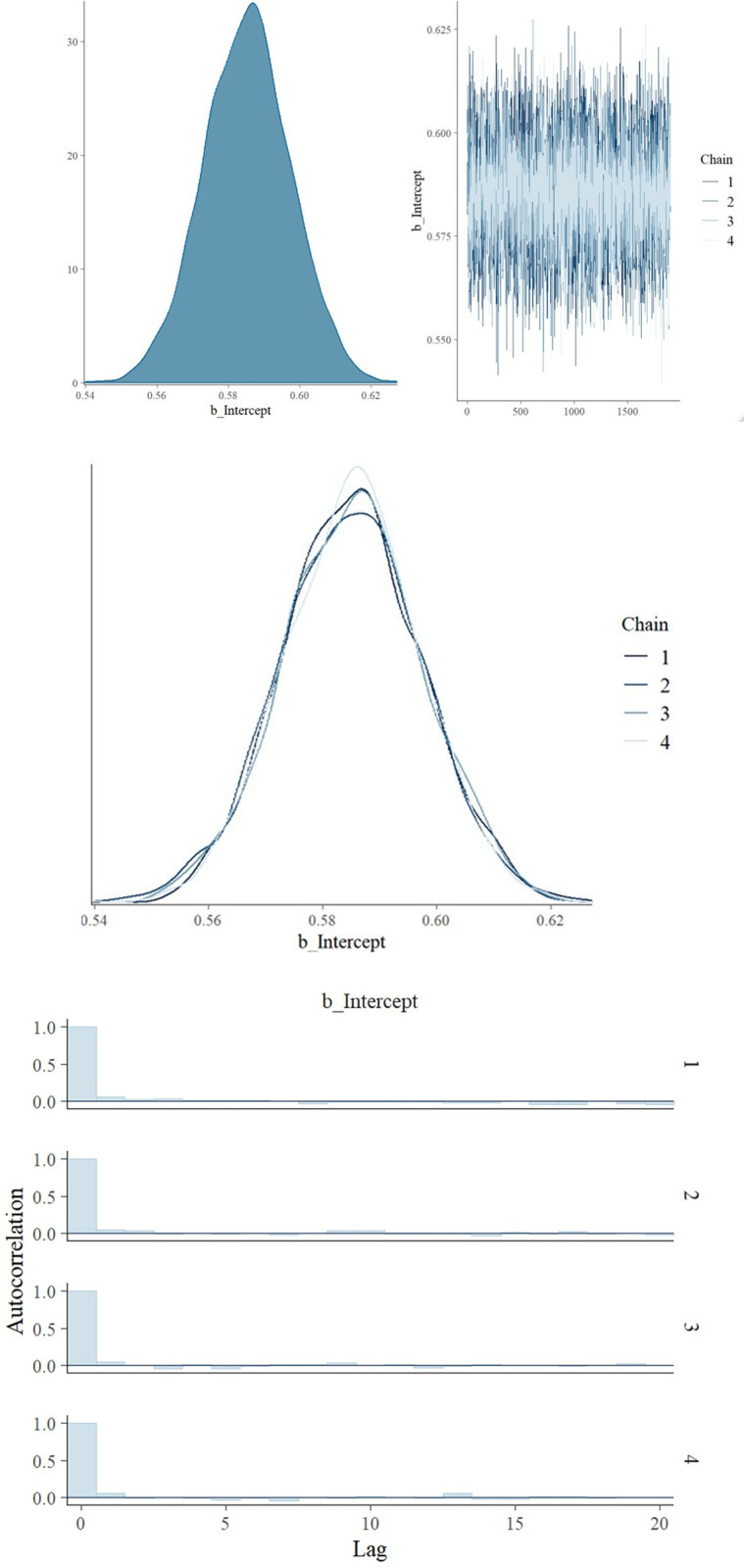
Trace (A), density (B), and autocorrelation (C) plots for primary analysis using prior from GENOA dataset. ***Abbreviations:*** GENOA: Genetics of Opioid Addiction study.

Fig 3 presents our assessment of convergence for the primary analysis, through trace *(A)*, density *(B)*, and autocorrelation *(C)* plots. Our trace plot demonstrates well-mixed chains, whereas the density plot depicts a unimodal and symmetric distributions, both of which indicate convergence. The autocorrelation plots in Fig 3C demonstrate weak autocorrelation, decreasing rapidly with lag, confirming good mixing and convergence of chains.

The R-hat values, and bulk and tail effective sample sizes for all analyses may be found in Table 4. R-hat values of 1.0 indicate convergence has been achieved for our primary and first two sensitivity analyses. We also observe bulk and tail effective sizes of greater than 1,000, thereby suggesting reliability of our analyses.

Assessment of convergence for our non-informative and sensitivity analyses may be found in *Figs B to F in* S3 File *Supplementary tables and figs*. Convergence assessment for our non-informative and first two sensitivity analyses were similar to that of our primary analysis. However, in our third sensitivity analysis, R-hat values reached 1.05, accompanied by low effective sample sizes, implying unreliable estimates. This lack of convergence was further evidenced by poor mixing of Markov chains in the trace plots, non-overlapping density plots, and unstable autocorrelation patterns (*Fig E in* S3 File *Supplementary tables and figs*). Conversely, in our fourth sensitivity analysis, we observed improved mixing compared to the primary analysis. The Markov chains smoothly explored the parameter space, with rapid transitions between points. Density plots showed overlapping chains, indicating convergence. Moreover, autocorrelation plots displayed weaker autocorrelation, decreasing rapidly with lag, signifying improved mixing and strong convergence (*Fig F in* S3 File *Supplementary tables and figs*).

## Discussion

Our findings suggest that there is a 95% probability that the true prevalence of mood and/or anxiety disorders among people with OUD lies between 56.0 and 61.0%, with an estimated mean prevalence of 59.0%. A similar prevalence of mood and/or anxiety disorders has been obtained by a retrospective cohort study. [106]

Our analysis considered both responses to self-reported diagnoses obtained from GENOA study participants and administrative health diagnoses from ICES databases, and we obtained similar estimates from our sensitivity analyses suggesting the robustness of our findings. Our posterior prevalence estimate resides between the prevalence estimates obtained from the use of a self-reported diagnostic tool (56.6%) and administrative health records (59.6% for our primary analysis), which was similarly observed by another Ontario-based study that used a Bayesian approach to assess prevalence of these mental health disorders across different sources. [5] Furthermore, our estimate is closer to that obtained from our prior, i.e., administrative health records, owing to the larger sample size of this data source. However, our overall prevalence estimates are quite similar across both data sources.

There are two potential reasons for the unexpected concordance between prevalence of mood and/or anxiety disorders in self-report and administrative health records. First, although discordance has been observed by other studies, [1–3,5,69] people with OUD access health services at a higher rate than those without OUD. [107] Thus, administrative health records may adequately capture their health service use for treatment of mood and/or anxiety disorders, which are otherwise underreported. [1,2,6,69] Second, our ICES-derived diagnoses encompassed a broader spectrum of psychiatric conditions than the MINI. For instance, conditions like dysthymic and adjustment disorders were included in our administrative-level data, but not captured by the MINI in the GENOA data. While this definition aligns with that used by other studies, [85] it resulted in an omnibus mental health condition in our analysis, potentially leading to a more inflated prevalence estimate than would otherwise have been obtained in administrative health records. [5,90]

### Strengths and limitations

Our objective was to provide a combined estimate of mood and/or anxiety disorders among people with OUD, using data from a self-reported diagnostic tool and administrative health records. Although prior studies have examined prevalence of these disorders separately, concurrent assessment allows for coordination of care, particularly in populations with high rates of psychiatric comorbidities such as those with OUD. [82–84] Furthermore, if there was greater discordance between prevalence estimates, i.e., potentially through using different priors, a Bayesian analysis would be able to provide a more informative assessment of prevalence. However, it is important to acknowledge that Bayesian methods may require more computational resources and trained personnel compared to frequentist methods, which may limit their accessibility for some research teams. Given that self-report diagnostic tools and administrative data capture patients across a range of severities and treatment stages, [2,3] combining them provides an estimate reflecting a wider spectrum of common psychiatric disorders in the population. Thus, we believe our tutorial example serves useful to mental health researchers assessing various psychiatric or physical health conditions which may have different prevalence estimates across diverse data sources.

To our knowledge, ours is the first study to combine data obtained from a self-reported diagnostic tool with administrative health records to assess the prevalence of mood and/or anxiety disorders among people with OUD and we followed rigorous methodology used by prior research. However, our study is not without its limitations. First, our analyses are limited by the accuracy of the measurement instruments, i.e., the MINI and administrative-derived diagnoses. In particular, we lacked information on validity of administrative-derived diagnoses for anxiety disorders, with validity estimates from other jurisdictions for MDD diagnoses in administrative health records. It is important to note that incorporation of the sensitivity and specificity of each measure, via a Bayesian analysis, as conducted by a previous Ontario linkage study, [5] would have enabled us to integrate information regarding psychometric properties and concordance between these measures. However, due to the small sample of GENOA participants who completed the MINI, the resulting sample (*n* = 549) would have been smaller than that used in our analysis (*n* = 1,061), and we therefore opted to use our methodological approach. Future linkage studies with larger sample sizes should integrate psychometric properties of measures, in order to provide a more accurate estimate of prevalence. Second, we used a prior from health records due to our aim in assessing potential discordance between estimates obtained from self-reported diagnostic tools and administrative data. However, use of more evidence-informed priors, i.e., as obtained through meta-analyses, is recommended for Bayesian analyses. Although a meta-analysis estimate was not available for our combined prevalence of mood and/or anxiety disorders, authors of future studies assessing occurrence of single psychiatric comorbidities among OUD populations should refer to the recent meta-analysis conducted on this topic. [59] This is particularly applicable when assessing anxiety disorders, for which there is limited evidence regarding validation of administrative-derived diagnoses. [5] Third, findings from our study may be limited, as we only examined people with OUD in Ontario, and research has documented nation-wide differences in guidelines for opioid agonist detection and treatment, which may result in differences in populations. [108]

### Tutorial overview: Lessons learned

Although findings from our Bayesian and frequentist analyses are similar overall, we would like to highlight the key lessons learned from our tutorial.

1. There are differences when interpreting prevalence estimates and confidence/credible intervals from frequentist and Bayesian analyses, respectively, with only Bayesian methods being able to provide direct probability statements about estimated parameters.
2. Non-informative priors in Bayesian analyses result in similar findings as frequentist analyses, a finding which is supported by empirical research. [109]
3. When estimates obtained from informative priors are consistent with study data, the results of Bayesian and frequentist analyses are similar in magnitude. [110]
4. Use of informative priors results in narrower and taller posterior distributions and, consequently, narrower credible intervals as compared to frequentist confidence intervals.
  Note: This was observed when we varied our priors from our main to sensitivity analyses, the latter of which used larger samples, and therefore, resulted in greater certainty of our estimated parameter.
5. Informative priors, which are inconsistent with study data may influence the results of Bayesian analyses. Although this was not observed in our tutorial example, this has been demonstrated by prior research. [110] Thus, researchers must be judicious when selecting a prior for their studies.
6. Use of an adequate number of iterations is necessary to achieve convergence. Convergence should be confirmed by checking the appropriate trace, density, and autocorrelation plots, in addition to R hat values, and bulk tail effective sample sizes.

## Guidance for reporting findings from Bayesian analyses

There should be considerable attention paid to the reporting of epidemiological research, which uses Bayesian analyses. A recent review has demonstrated that reporting of Bayesian analysis is inadequate, with particular note for the incompleteness of methods and results sections. Furthermore, the authors of this empirical study observed a lack of specification and evaluation related to the prior used in these studies. [111]

In order to facilitate transparent reporting of Bayesian analyses, we encourage researchers to consult the guidance suggested on the Enhancing the QUAlity and Transparency Of health Research (EQUATOR) network (https://www.equator-network.org/?s=bayesian&submit=Go). These include the ROBUST criteria for reporting Bayesian analyses, [21] in addition to JASP, [22] BayesWatch [112] and Bayesian Analysis Reporting Guidelines (BARG). [23] In our tutorial, we used the ROBUST criteria for reporting our analysis, which may be found in S2 File ROBUST checklist. Given the simplicity of our analysis, this guideline was appropriate and included the following seven items:

1. **Methods:** Prior distribution – (i) specified, (ii) justified, (iii) sensitivity analysis.
2. **Methods:** Analysis – (iv) statistical model, (v) analytic technique.
3. **Results:** (vi) central tendency, (vii) standard deviation or credible interval.

Adherence to more comprehensive reporting guidelines, i.e., the BARG, which subsumes the ROBUST criteria, [23] is recommended for more complex analyses. However, at the very least, authors should report the aforementioned seven items when reporting findings from Bayesian analyses in their research. We additionally encourage researchers to include checklists with their submitted manuscripts, which has been demonstrated to improve adherence to reporting guidelines. [113]

### Concluding remarks

Bayesian analysis offers a framework for integrating information from multiple data sources to estimate population-level parameters with increased accuracy. This may be particularly useful in psychiatric epidemiology research, where there is discordance between estimates obtained across a range of data sources. It is additionally important to highlight that Bayesian statistics are not intended to be viewed as inherently superior to frequentist methods. Rather, researchers should view both approaches as part of a broader spectrum of methodological choices, each with its own set of advantages and disadvantages. Bayesian approaches align with many aspects of clinical research and evidence-informed decision-making, where prior information is often utilized to inform current practices and choices. Additionally, Bayesian statistics is beneficial in its ability to provide direct probability statements about estimated parameters. Mental health researchers opting to use Bayesian methods for estimating prevalence should clearly define and justify the choice of priors, and assess the robustness of their findings through sensitivity analysis. Authors should adhere to reporting guidelines, and include full documentation of the statistical model and findings from their Bayesian analysis.

## Supporting information

S1 File STROBE checklist. (DOCX)

S2 File ROBUST checklist. (DOCX)

S3 File Supplementary tables and figures. (DOCX)

S4 File R script. (R)

S5 File R script. (DOCX)

S6 File JASP guide. (DOCX)
