# Supplementary material for: A tutorial on the what, why, and how of Bayesian analysis: Estimating mood and anxiety disorder prevalence using a Canadian data linkage study
Source: PLOS Ment Health. 2025 Feb 26;2(2):e0000253. doi: 10.1371/journal.pmen.0000253 (PMC12798518; doi:10.1371/journal.pmen.0000253)
Supplement: S2 File — (DOCX) [file pmen.0000253.s002.docx]

S2 File – ROBUST Reporting Criteria—Recommended items that should be included in Bayesian analyses of clinical studies

A tutorial on the what, why, and how of Bayesian analysis: estimating mood and anxiety disorder prevalence using a Canadian data linkage study

|  | Item No | Recommendation | Page No |
| --- | --- | --- | --- |
| **Prior distribution** |  | | |
|  | 1 | Specified | 23-24, Table 2 |
|  | 2 | Justified | 21, 23 |
|  | 3 | Sensitivity analysis | 25-26, Table 2 |
| **Analysis** | | | |
|  | 4 | Statistical model | 23 |
|  | 5 | Analytical technique | 23 |
| **Results** |  |  | |
|  | 6 | Central tendency | 29-30, Table 4 |
|  | 7 | Standard deviation or credible interval | 29-30, Table 4 |

***Note:*** This is not a formal checklist but a recommended list of items based on the following publication:

Sung L, Hayden J, Greenberg ML, Koren G, Feldman BM, Tomlinson GA. Seven items were identified for inclusion when reporting a Bayesian analysis of a clinical study. *Journal of clinical epidemiology.* 2005 Mar 1;58(3):261-8.
