# Supplementary material for: A tutorial on the what, why, and how of Bayesian analysis: Estimating mood and anxiety disorder prevalence using a Canadian data linkage study
Source: PLOS Ment Health. 2025 Feb 26;2(2):e0000253. doi: 10.1371/journal.pmen.0000253 (PMC12798518; doi:10.1371/journal.pmen.0000253)

S3 File – Supplementary tables and figures

A tutorial on the what, why, and how of Bayesian analysis: estimating mood and anxiety disorder prevalence using a Canadian data linkage study

Contents

[Fig A: Study flow diagram for ICES linked database 2](#_Toc189136688)

[Table A: ICES billing codes for mood and anxiety disorders 3](#_Toc189136689)

[Fig B: Trace (i), density (ii), and autocorrelation (iii) plots using non-informative β(1,1) prior 4](#_Toc189136690)

[Fig C: Trace (i), density (ii), and autocorrelation (iii) plots for sensitivity analysis 1 using prior from POST dataset 5](#_Toc189136691)

[Fig D: Trace (i), density (ii), and autocorrelation (iii) plots for sensitivity analysis 2 using prior from POST + GENOA merged dataset 6](#_Toc189136692)

[Fig E: Trace (i), density (ii), and autocorrelation (iii) plots for sensitivity analysis 3 - decreasing iterations to assess convergence 7](#_Toc189136693)

[Fig F: Trace (i), density (ii), and autocorrelation (iii) plots for sensitivity analysis 4 - increasing iterations to assess convergence 8](#_Toc189136694)

# Fig A: Study flow diagram for ICES linked database

Final GENOA and POST Cohort, with duplicates excluded

*n* = 3,486

Exclusion due to invalid linkage to ICES holdings

*n* = 23

Exclusion due to death date before index

*n* = 8

Exclusion due to no OHIP eligibility or out of Province at index

*n* = 25

Study sample successfully linked with ICES holdings and in analysis

*n* = 3,430

*(GENOA: n = 1,061*

*POST: n = 2,369)*

GENOA cohort: *n* = 1,333

*(MINI subset: n= 549,*

*other participants: n = 784)*

POST cohort: *n*= 2,425

Merged study sample: *n* = 3,758

Exclusion due to duplicate enrolment

*n* = 272

# Table A: ICES billing codes for mood and anxiety disorders

| **Billing codes for mood disorders** | **Databases** |
| --- | --- |
| ***1 hospital discharge (CIHI-DAD) or 2 physician visits (OHIP) within a 2-year period***  **ICD-9/OHIP:** 296, 300, 309, 311  **ICD-10:** F30-F33, F341, F348, F349, F38-F42, F431, F432, F438, F44, F45.0, F45.1, F45.2, F48, F530, F68.0, F930, F99 | DAD, OHIP, OMHRS |
|  |  |
|  |  |
|  |  |
|  |  |

***Abbreviations:*** CIHI-DAD: Canadian Institute for Health Information Discharge Abstract Database, ICD: International Classification of Diseases (Ninth and Tenth Revisions), OHIP: Ontario Health Insurance Plan, OMHRS: The Ontario Mental Health Reporting System.

# Fig B: Trace (i), density (ii), and autocorrelation (iii) plots using non-informative β(1,1) prior


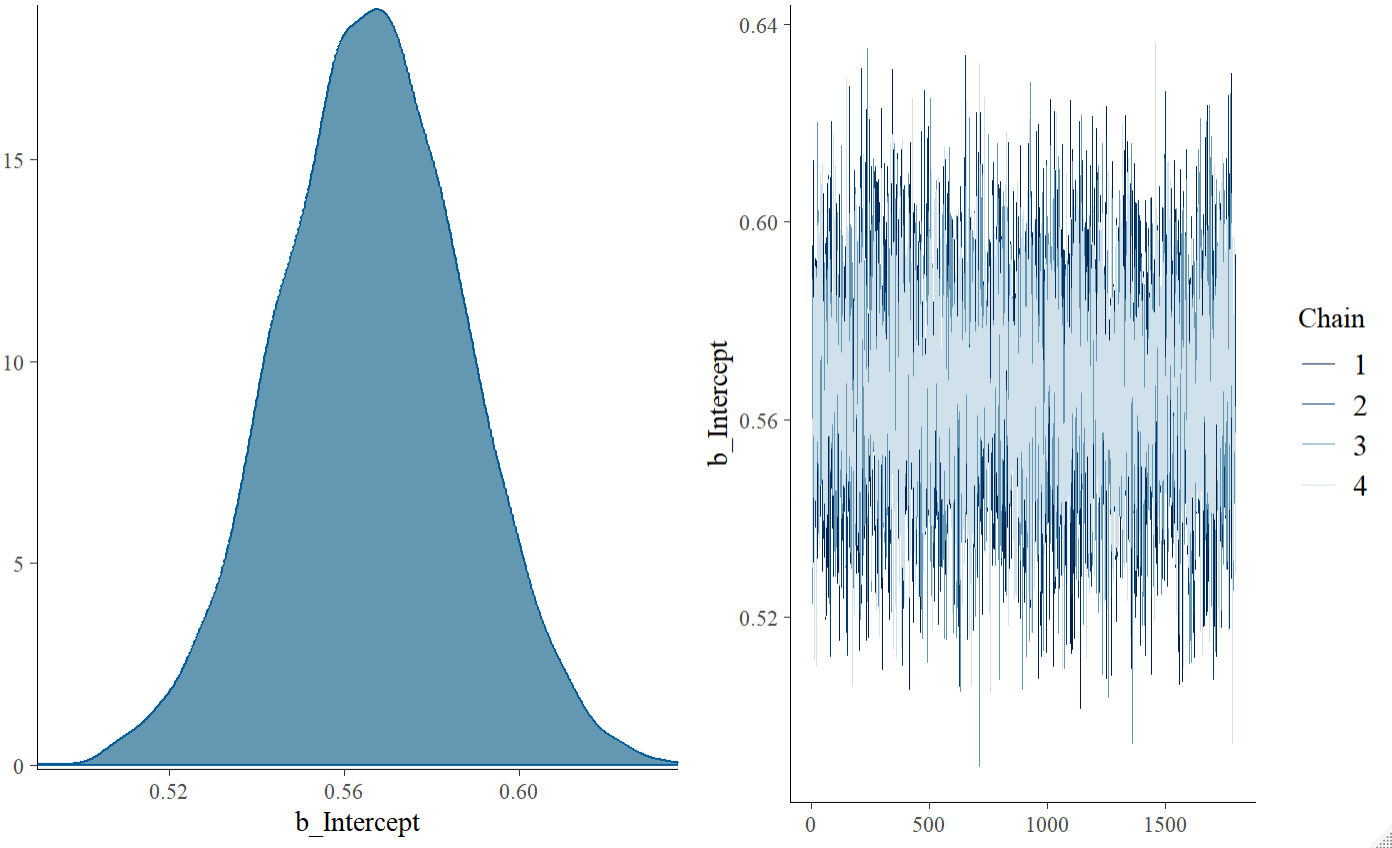


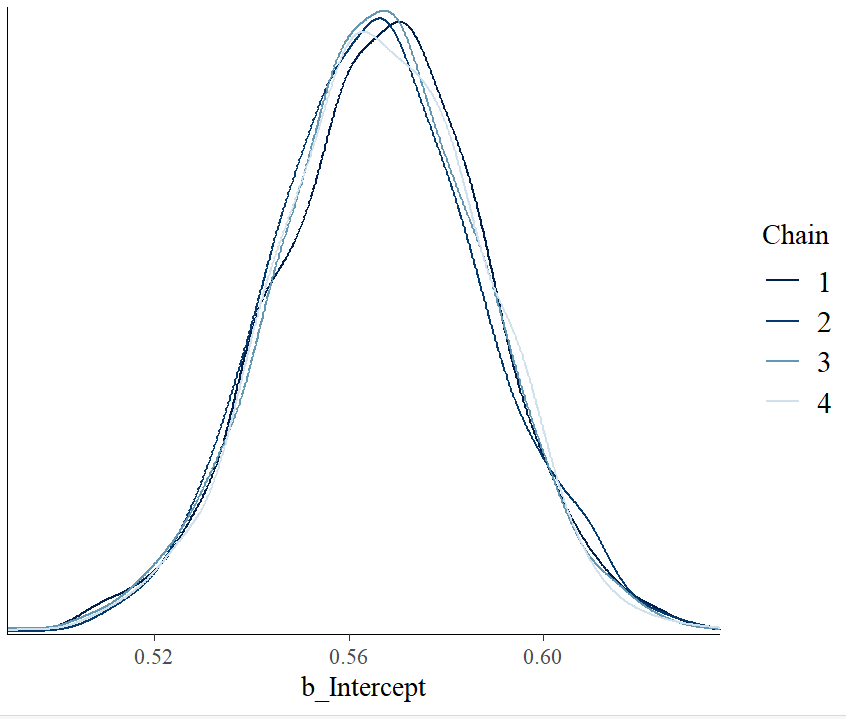


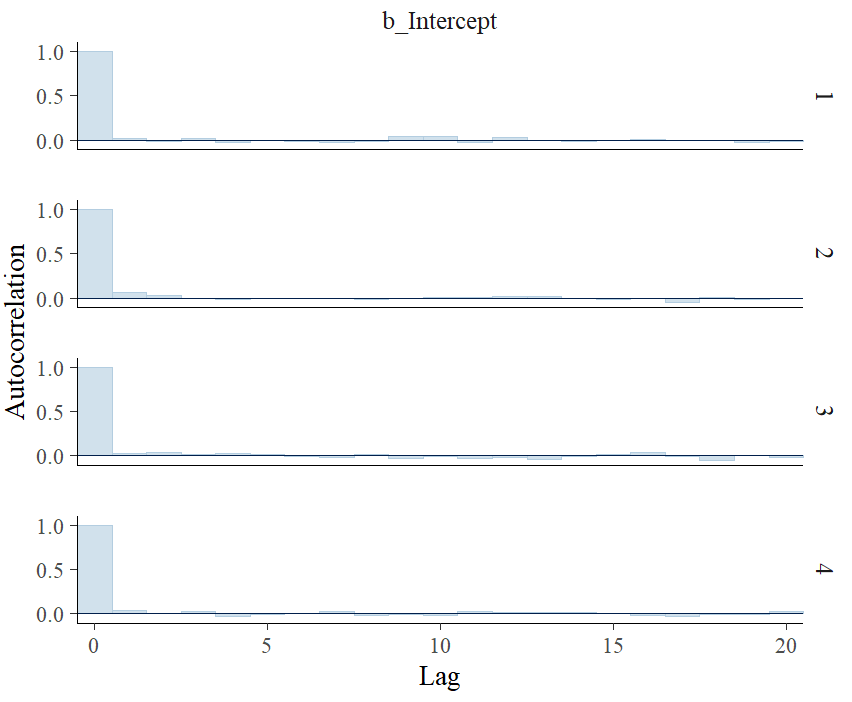


# Fig C: Trace (i), density (ii), and autocorrelation (iii) plots for sensitivity analysis 1 using prior from POST dataset


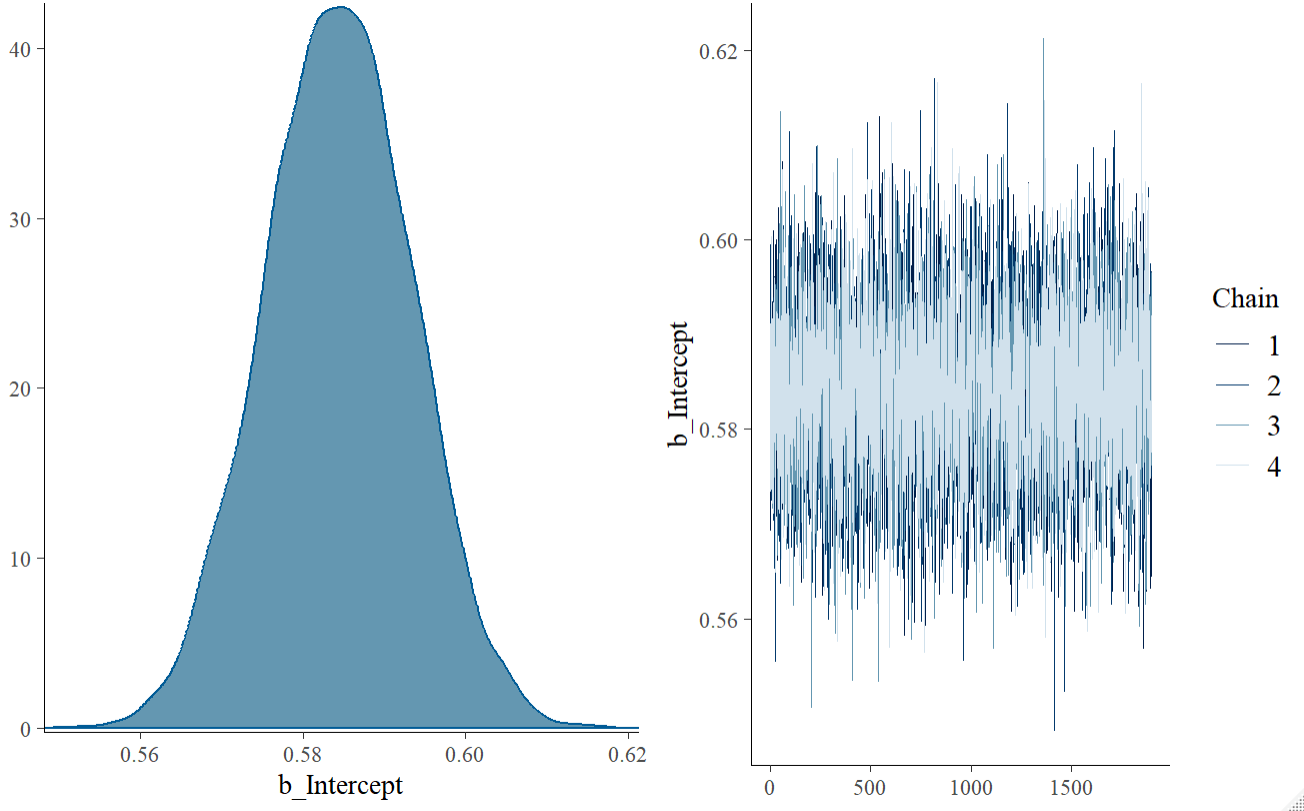


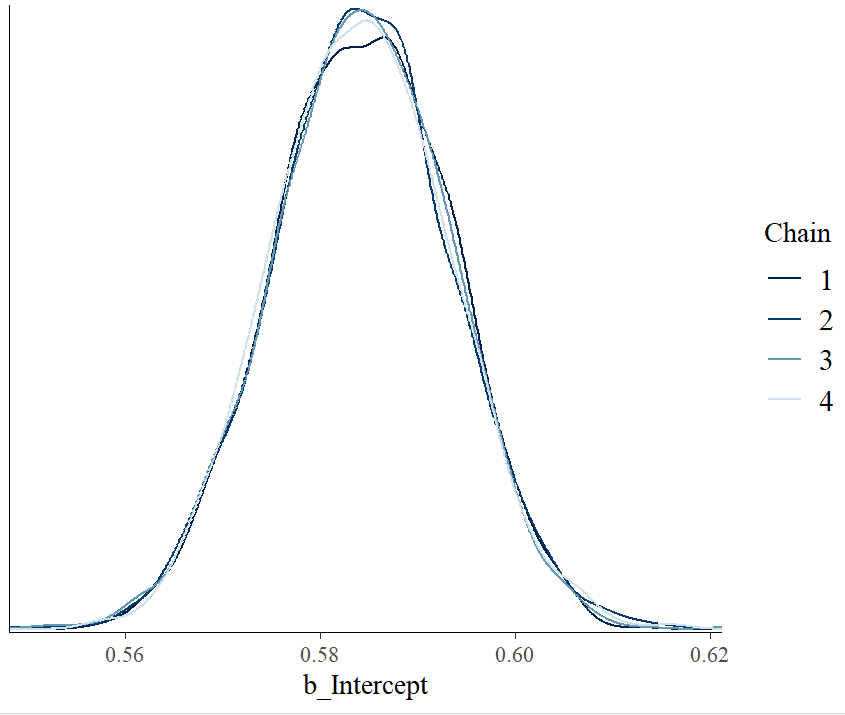


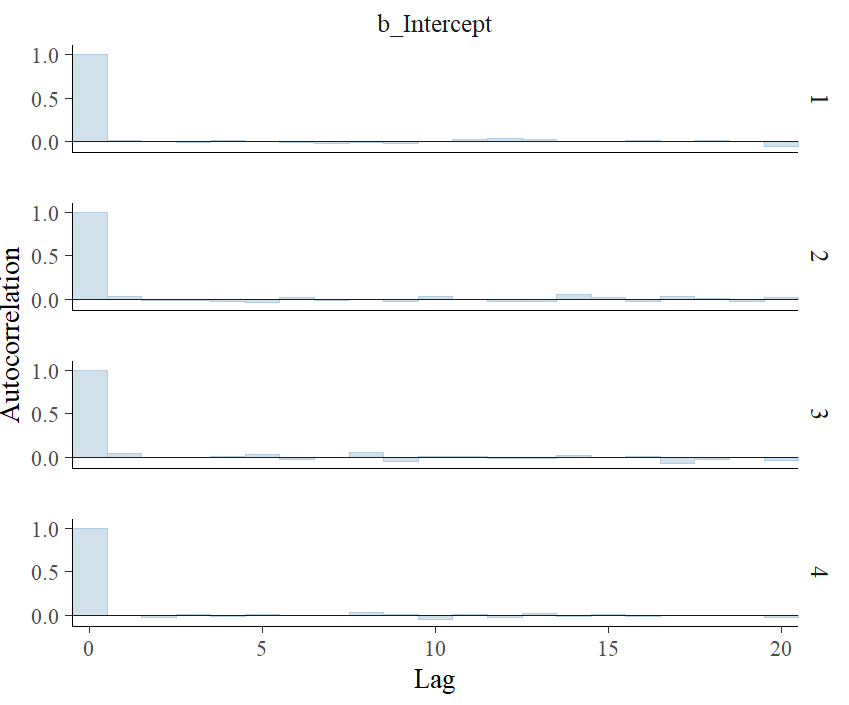


# Fig D: Trace (i), density (ii), and autocorrelation (iii) plots for sensitivity analysis 2 using prior from POST + GENOA merged dataset


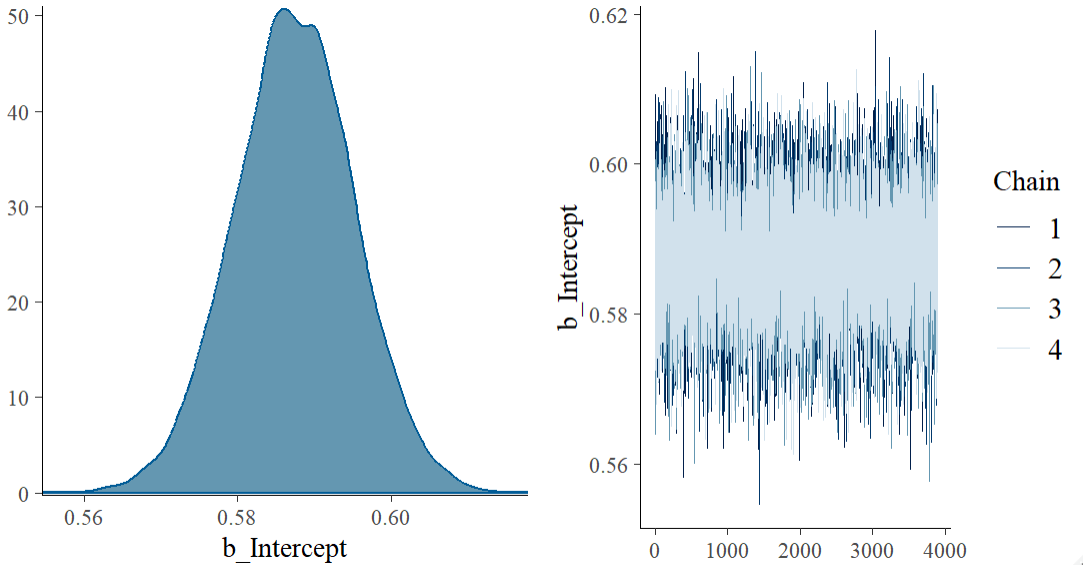


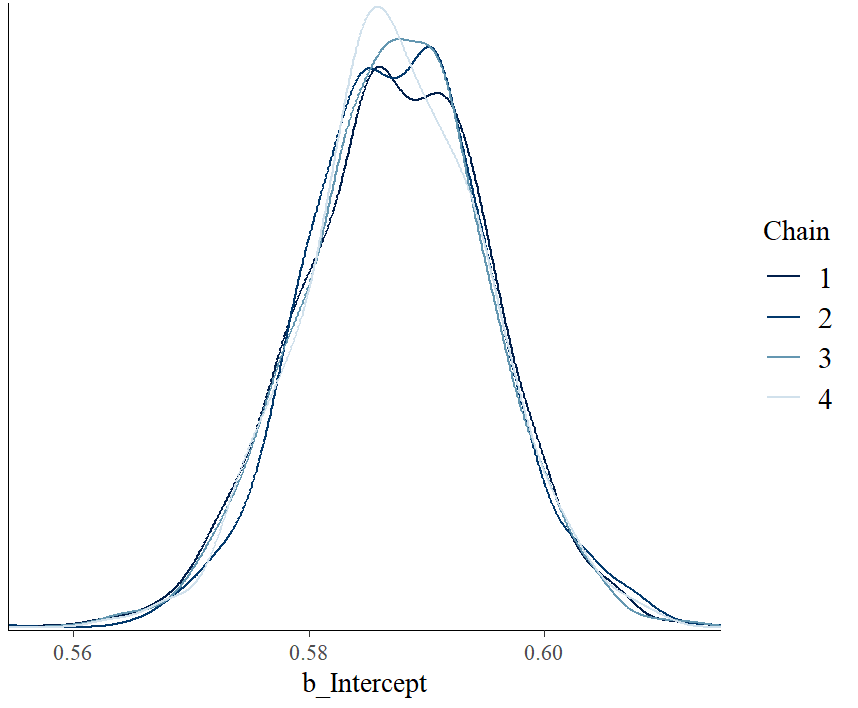


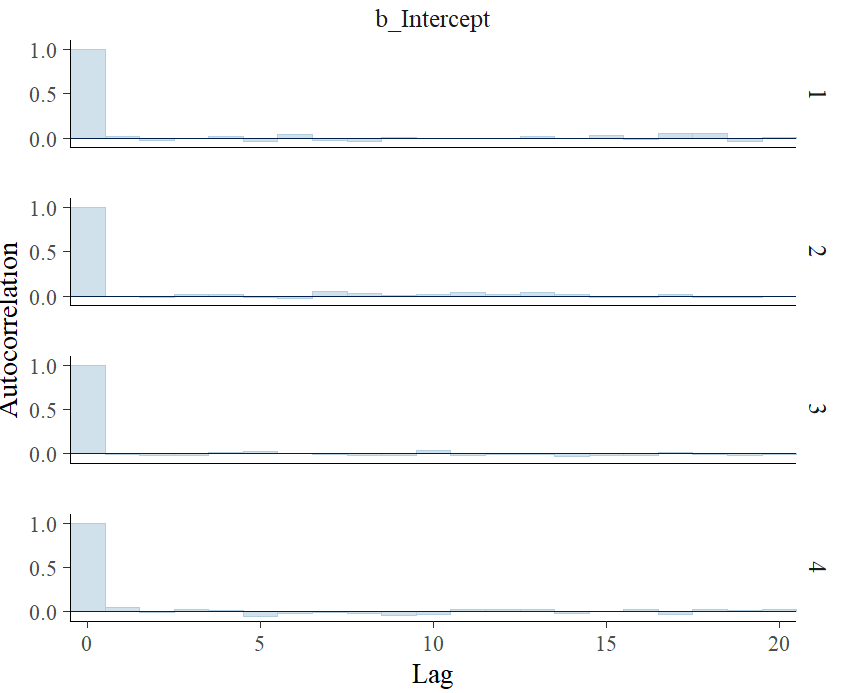


# Fig E: Trace (i), density (ii), and autocorrelation (iii) plots for sensitivity analysis 3 - decreasing iterations to assess convergence


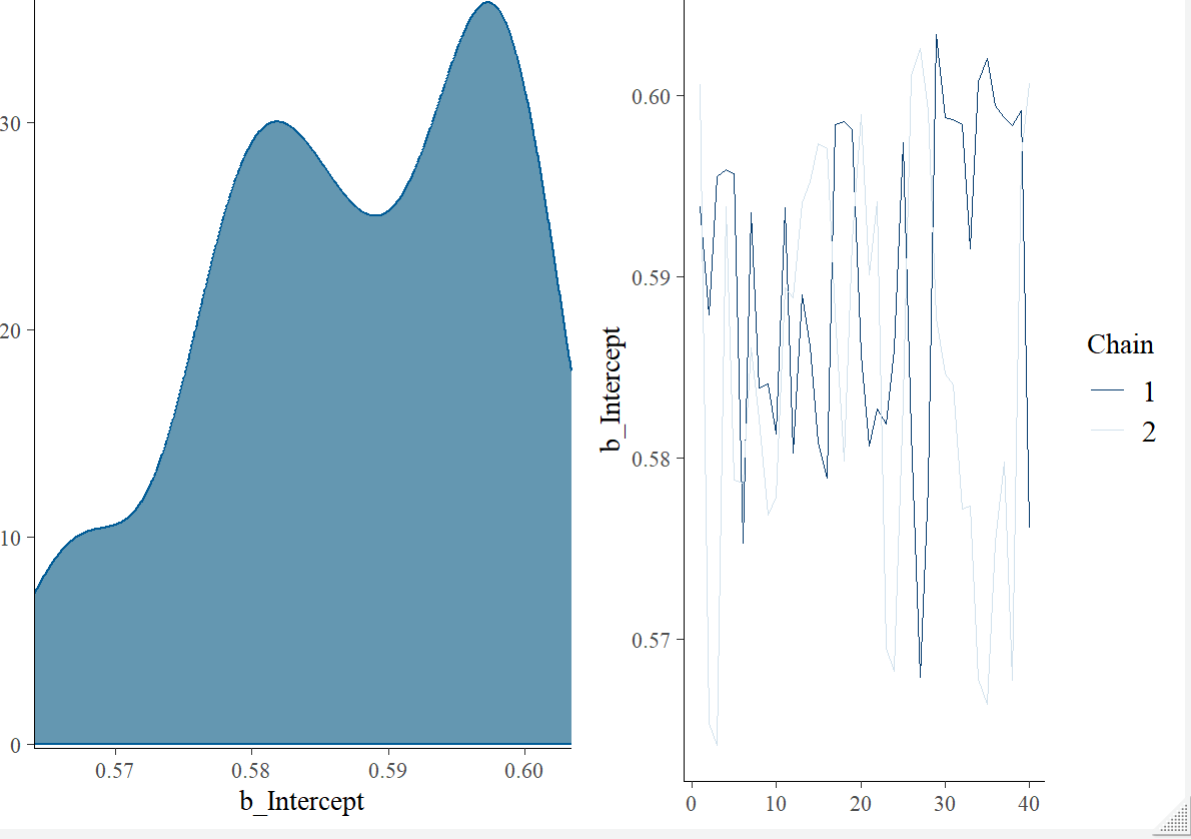


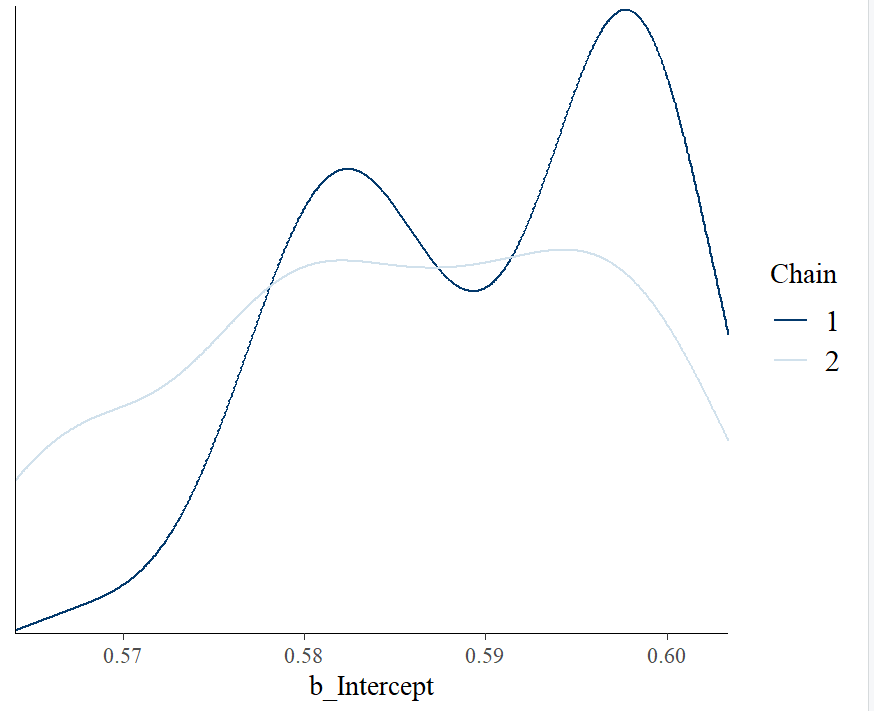


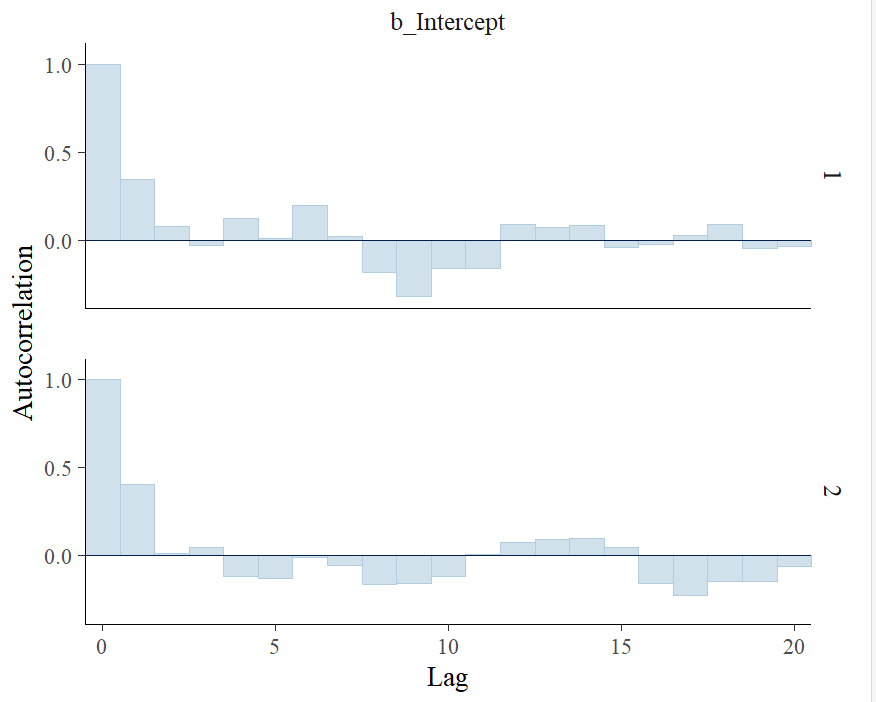


# Fig F: Trace (i), density (ii), and autocorrelation (iii) plots for sensitivity analysis 4 - increasing iterations to assess convergence


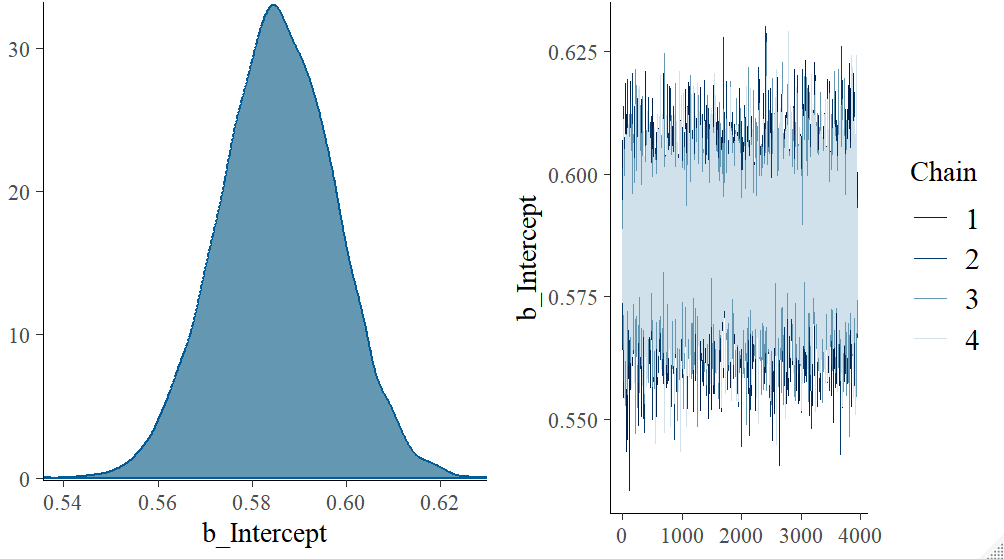


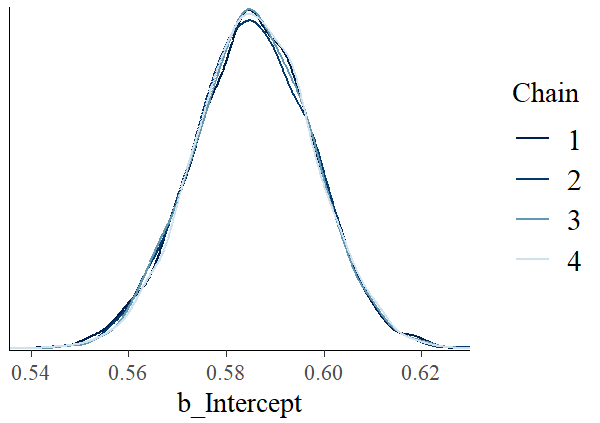


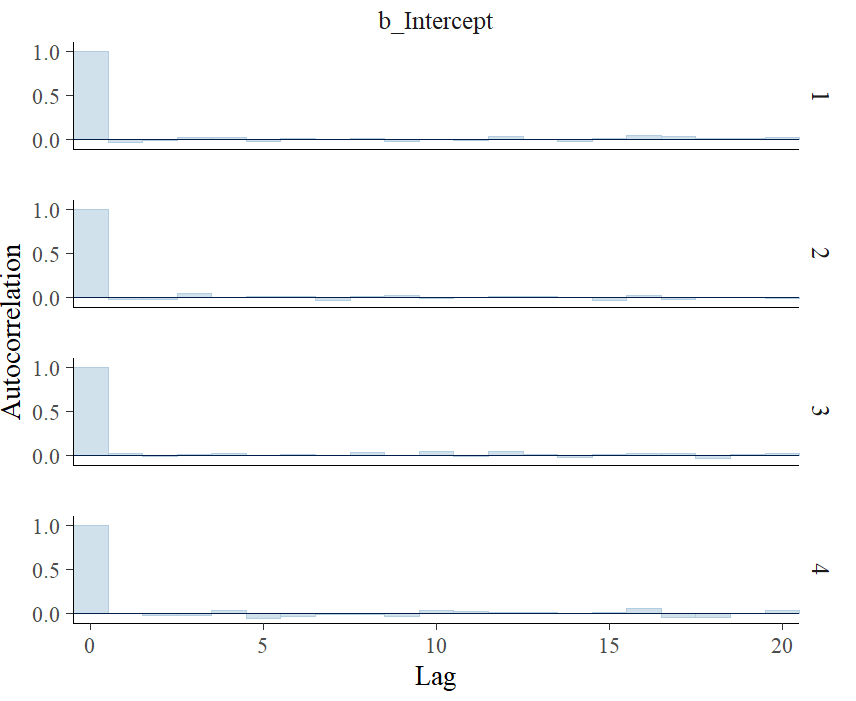

Supplement: S3 File — (DOCX) [file pmen.0000253.s003.docx]
