# Supplementary material for: A tutorial on the what, why, and how of Bayesian analysis: Estimating mood and anxiety disorder prevalence using a Canadian data linkage study
Source: PLOS Ment Health. 2025 Feb 26;2(2):e0000253. doi: 10.1371/journal.pmen.0000253 (PMC12798518; doi:10.1371/journal.pmen.0000253)
Supplement: S5 File — (DOCX) [file pmen.0000253.s005.docx]

S5 File – R script

A tutorial on the what, why, and how of Bayesian analysis: estimating mood and anxiety disorder prevalence using a Canadian data linkage study

####################S4_File - R Script############################

setwd("~/PhD 2020-2024/5. Courses/Winter 2024/HRM 750 Bayesian design and analysis/Assignments/Final Assignment")

#### A tutorial on the what, why, and how of Bayesian analysis: estimating mood and anxiety disorder prevalence using a Canadian data linkage study

#loading packages

library(rstan)

library(brms)

library(ProbBayes)

library(tidyverse)

library(ggpubr)

library(bayesplot)

# load genoa dataset

d<-read.csv("Bayesian.csv")

head(d)

d

#rates of mood or anxiety in the genoa dataset - raw proportion

table(d$moodoranxiety)

311/549

#frequentist approach

fit_glm_freq <- glm(moodoranxiety ~ 1,

family = binomial(link = "identity"),

data = d)

summary(fit_glm_freq)

confint(fit_glm_freq)

########################fit 1 - non informative prior

# Bayesian with beta(1,1) prior

#we can set a seed to enhance replicability and include a certain number of iterations and warm-up draws with chains, we also need to thin out chains

#this will come into use later when we assess convergence

fit1 <- brm(data = d,

family = bernoulli(link = "identity"),

moodoranxiety ~ 1,

seed = 123,iter = 20000, warmup =500,chains = 4,thin = 5 )

summary(fit1)

#trace plot

plot(fit1)

# density overlay plot

mcmc_dens_overlay(fit1, pars = c("b_Intercept"))

#acf plot

mcmc_acf_bar(fit1, pars = c("b_Intercept"))

########################fit 2 - primary analysis

# Bayesian with prior from genoa total dataset

fit2 <- brm(data = d,

family = bernoulli(link = "identity"),

moodoranxiety ~ 1,

prior = c(prior(beta(633, 430), class = Intercept, lb=0.5)),

seed = 123,iter = 20000, warmup =500,chains = 4,thin = 5 )

summary(fit2)

#trace plot

plot(fit2)

# density overlay plot

mcmc_dens_overlay(fit2, pars = c("b_Intercept"))

#acf plot

mcmc_acf_bar(fit2, pars = c("b_Intercept"))

########################fit 3 - sensitivity analysis 1

# Bayesian with prior from post dataset

fit3 <- brm(data = d,

family = bernoulli(link = "identity"),

moodoranxiety ~ 1,

prior = c(prior(beta(1395, 976), class = Intercept, lb=0.5)),

seed = 123,iter = 20000, warmup =500,chains = 4,thin = 5 )

summary(fit3)

#trace plot

plot(fit3)

# density overlay plot

mcmc_dens_overlay(fit3, pars = c("b_Intercept"))

#acf plot

mcmc_acf_bar(fit3, pars = c("b_Intercept"))

########################fit 4 - sensitivity analysis 2

# Bayesian with prior from total merged (genoa + post) dataset

fit4 <- brm(data = d,

family = bernoulli(link = "identity"),

moodoranxiety ~ 1,

prior = c(prior(beta(2027, 1405), class = Intercept, lb=0.5)),

seed = 123,iter = 20000, warmup =500,chains = 4,thin = 5 )

summary(fit4)

#trace plot

plot(fit4)

# density overlay plot

mcmc_dens_overlay(fit4, pars = c("b_Intercept"))

#acf plot

mcmc_acf_bar(fit4, pars = c("b_Intercept"))

########################- convergence and sampling diagnostics

####fit 5 - - sensitivity analysis 3 - decrease iterations, warmup less, use less chains, thin out less

fit5 <- brm(data = d,

family = bernoulli(link = "identity"),

moodoranxiety ~ 1,

prior = c(prior(beta(633, 430), class = Intercept, lb=0.5)),

seed = 123,iter = 50, warmup =10,chains = 2,thin = 1 )

summary(fit5)

#trace plot

plot(fit5)

# density overlay plot

mcmc_dens_overlay(fit5, pars = c("b_Intercept"))

#acf plot

mcmc_acf_bar(fit5, pars = c("b_Intercept"))

####fit 6 - - sensitivity analysis 4- increase iterations, warmup more, thin out more

fit6 <- brm(data = d,

family = bernoulli(link = "identity"),

moodoranxiety ~ 1,

prior = c(prior(beta(633, 430), class = Intercept, lb=0.5)),

seed = 123,iter = 40000, warmup =600,chains = 4,thin = 10)

summary(fit6)

#trace plot

plot(fit6)

# density overlay plot

mcmc_dens_overlay(fit6, pars = c("b_Intercept"))

#acf plot

mcmc_acf_bar(fit6, pars = c("b_Intercept"))

#####################################################end
