# Supplementary material for: A tutorial on the what, why, and how of Bayesian analysis: Estimating mood and anxiety disorder prevalence using a Canadian data linkage study
Source: PLOS Ment Health. 2025 Feb 26;2(2):e0000253. doi: 10.1371/journal.pmen.0000253 (PMC12798518; doi:10.1371/journal.pmen.0000253)
Supplement: S6 File — (DOCX) [file pmen.0000253.s006.docx]

S6 File – JASP guide

A tutorial on the what, why, and how of Bayesian analysis: estimating mood and anxiety disorder prevalence using a Canadian data linkage study

**Step 1:** Download JASP from the following webpage: <https://jasp-stats.org/>.

**Step 2:** Load dataset into JASP.

**Step 3:** Select Frequencies 🡪 Bayesian Binomial Test.

**Step 4:** Select the outcome variable (mood or anxiety disorders), and input priors (α=633, β=430 for primary analysis).

**Step 5:** The plots, with the posterior median prevalence, will be generated.

**Step 6:** R syntax will also be generated:

*jaspFrequencies::BinomialTestBayesian(*

*version = "0.18.3",*

*formula = ~ moodoranxiety,*

*priorA = "633",*

*priorB = "430",*

*priorPosteriorPlot = TRUE)*

**Step 7:** The priors may be varied in the R syntax window or inputting the priors in the specified textbox.
